# Experimental characterisation of porcine subcutaneous adipose tissue under blunt impact up to irreversible deformation

**DOI:** 10.1007/s00414-021-02755-0

**Published:** 2021-12-04

**Authors:** Felicitas Lanzl, Fabian Duddeck, Saskia Willuweit, Steffen Peldschus

**Affiliations:** 1 Biomechanics and Accident Analysis, Institute of Legal Medicine, Ludwig-Maximilians-Universität München, Nussbaumstr. 26, 80336 Munich, Germany; 2 School of Engineering and Design, Technical University of Munich TUM, Arcisstr. 21, 80333 Munich, Germany

**Keywords:** Subcutaneous adipose tissue, Blunt force trauma, Experimental characterisation, Drop tower tests, Irreversible deformation, Photogrammetry

## Abstract

A deeper understanding of the mechanical characteristics of adipose tissue under large deformation is important for the analysis of blunt force trauma, as adipose tissue alters the stresses and strains that are transferred to subjacent tissues. Hence, results from drop tower tests of subcutaneous adipose tissue are presented (i) to characterise adipose tissue behaviour up to irreversible deformation, (ii) to relate this to the microstructural configuration, (iii) to quantify this deformation and (iv) to provide an analytical basis for computational modelling of adipose tissue under blunt impact. The drop tower experiments are performed exemplarily on porcine subcutaneous adipose tissue specimens for three different impact velocities and two impactor geometries. An approach based on photogrammetry is used to derive 3D representations of the deformation patterns directly after the impact. Median values for maximum impactor acceleration for tests with a flat cylindrical impactor geometry at impact velocities of 886 mm/s, 1253 mm/s and 2426 mm/s amount to 61.1 g, 121.6 g and 264.2 g, respectively, whereas thickness reduction of the specimens after impact amount to 16.7%, 30.5% and 39.3%, respectively. The according values for tests with a spherically shaped impactor at an impact velocity of 1253 mm/s are 184.2 g and 78.7%. Based on these results, it is hypothesised that, in the initial phase of a blunt impact, adipose tissue behaviour is mainly governed by the behaviour of the lipid inside the adipocytes, whereas for further loading, contribution of the extracellular collagen fibre network becomes more dominant.

## Introduction

Subcutaneous adipose tissue is the loose connective tissue located directly beneath the dermis of the skin. It represents one of the outer soft tissue layers of the human body. These layers are the first tissues to come into contact with a striking object during an impact event and, thus, alter the stresses and strains that are transferred to underlying tissues such as bones or internal organs. Therefore, a deeper understanding of the mechanical characteristics of adipose tissue is relevant for forensic biomechanics, especially for the investigation of blunt force trauma. Studies on different impact scenarios show that the superficial soft tissue layers can absorb between 35% up to nearly 70% of the impact energy [1–4]. However, the influence of the covering soft tissues on skeletal trauma, which can be essential for the reconstruction of blunt force attacks or the assessment of an assault as potentially life-threatening or not, is still not fully understood [5–11]. A promising tool for the evaluation of this influence is the finite element (FE) method, which has already been applied for the investigation of different impact scenarios in the field of forensic science [5, 12–16]. But for the application of these models, experimental data on the behaviour of the involved tissues is needed for the intended loading scenarios to develop and validate appropriate material descriptions, which can be quite challenging for soft tissues such as subcutaneous adipose tissue [16–19].

The principal structural elements of subcutaneous adipose tissue consist of a collagenous extracellular matrix surrounding lipid-filled cells, so-called adipocytes [20, 21]. Adipocytes exhibit a nearly spherical shape with a diameter of ca. 80 μm and their interior is almost completely occupied by a vacuole of triglyceride lipid [21, 22]. The outer cell membrane of adipocytes is encapsulated by a basement membrane made up of sheet-like collagen type IV, which is, in turn, surrounded by a mesh of collagen fibrils (mainly collagen type I, V and VI) with a wall thickness of around 2 μm [20, 23–26]. Together, the basement membrane, as well as the fibrillary mesh, builds a foam-like structure that encloses each adipocyte and represents one of the two distinct collagenous elements of the adipose tissue extracellular matrix [20, 21, 25]. In the work of Comley and Fleck [21, 27], this structure is termed *reinforced basement membrane*. This denotation will also be used in the present study.

Adipocytes are organised in small assemblies without clear boundaries called lobules [26]. These lobules are interwoven by a network of collagen type I fibres, which comprises the second main structure of the adipose tissue extracellular matrix [20, 24–26]. Fibres of this network are termed interlobular septa and can exhibit a diameter between 10 nm for one single collagen fibre up to 30 μm for a bundle of collagen fibres and a length of several millimetres [26]. The composition of the interlobular septa is comparable to that of collagen fibres in the dermis [20, 21]. In addition, the network of interlobular septa is also reinforced by a smaller proportion of elastin fibres, similar to the collagen network of the dermis, according to Alkhouli et al. [20]. Comley and Fleck state that the structure of the interlobular septa is similar to that of an open-cell foam, whereas the structure of the reinforced basement membrane resembles a closed-cell foam [21, 27]. In addition to adipocytes and collagenous as well as elastin elements, subcutaneous adipose tissue comprises nerves, blood vessels and hair follicles [24, 28]. Lipid constitutes 60–80 mass % of adipose tissue, 5–30 mass % are water and the remaining 2–3 mass % are composed of proteins [21].

The material behaviour of adipose tissue has been investigated in several studies under different loading conditions. Compressive behaviour was analysed using setups for uniaxial confined or unconfined compression or indentation testing for porcine and ovine subcutaneous adipose tissue, human abdominal tissue, human breast tissue, the human calcaneal fat pad and human plantar soft tissue [29–39]. Results show that adipose tissue behaves non-linear, exhibiting a J-shaped stress–strain response, typical for hyperelastic materials [27, 33, 36, 38]. Under quasi-static unconfined compression, the stress–strain response at low compressive strains is nearly linear, showing only a slight slope. For higher compressive strains around 25–30%; however, there is a sudden increase in stress leading to a steep gradient in the stress–strain curve [27].

Tensile behaviour of adipose tissue was investigated by uniaxial and biaxial tension tests on porcine subcutaneous adipose tissue as well as human abdominal and subcutaneous adipose tissue [20, 27, 40]. Comley and Fleck report that the behaviour of porcine subcutaneous adipose tissue in uniaxial tension is nearly symmetric to the behaviour in uniaxial compression [27]. For human adipose tissue, also a non-linear, J-shaped stress–strain behaviour was observed for uniaxial as well as biaxial tensile experiments [20, 40]. Alkhouli et al. tested human subcutaneous and omental adipose tissue up to a tensile strain of 30% [20]. They found an initial modulus (defined as tangent modulus at zero strain) of around 1.6 kPa and an ultimate modulus (defined as tangent modulus at 30% strain) of around 11.7 kPa for human subcutaneous adipose tissue. This is comparable to the results of Comley and Fleck, who report an elastic modulus (tangent modulus at a strain of 10%) of around 1 kPa for porcine subcutaneous adipose tissue tested under quasi-static tension [27]. The study of Sommer et al. is the only published work, to our knowledge, presenting results from tensile testing of isolated adipose tissue until failure [40]. For human abdominal adipose tissue under biaxial tension, they report values of 10 kPa–13 kPa for ultimate tensile stress and a value of 1.21 for the maximal tissue stretch before failure.

Other studies assessed the material behaviour of porcine subcutaneous and human abdominal adipose tissues under shear [31, 40–43]. The relation between shear strain and shear stress is non-linear for strains above 0.1% and seems to exhibit a dependency on loading history [40–42]. Results of Sommer et al. suggest that, with rising shear deformation, adipose tissue exhibits strain-softening behaviour [40].

Some of the aforementioned studies also investigate the influence of loading velocity on adipose tissue behaviour [27, 30, 32, 33, 36, 40, 42]. Stress–strain response of adipose tissue seems to be strain rate dependent with higher loading velocities resulting in stiffer material behaviour. Comley and Fleck observed that adipose tissue behaviour under compression is not influenced by testing velocity for strain rates up to 10 s^−1^, whereas stress–strain response becomes strongly dependent on loading velocity for strain rates above this regime [27]. A similar behaviour can be found in studies of Gefen and Haberman and Miller-Young et al. [32, 36]. Sommer et al. observed that, for human abdominal tissue tested under biaxial tension, stress increased about 50% for 10 times higher loading velocity and 100% for a 20 times higher loading velocity compared to the normal testing speed of 4 mm/min [40].

Reports on the anisotropic behaviour of adipose tissue are controversial. Miller-Young et al. tested human calcaneal fat pads under compression in different orientations and could not find any direction dependence [36]. In contrast, Sommer et al. observed anisotropic behaviour for human abdominal adipose tissue tested under biaxial tension and triaxial shear [40]. They state that adipose tissue exhibits anisotropic behaviour due to a certain orientation of the interlobular septa within the adipose tissue [40]. Nevertheless, they did not observe distinct anisotropic behaviour for all tested specimens [40].

Despite these extensive studies on adipose tissue mechanics, some physical effects and the corresponding material characteristics still require further investigations. Proposing a computational model, Naseri et al. identified a lack of experimental data in a testing regime combining large deformation and high testing velocities [44]. Comley and Fleck examined the uniaxial compressive behaviour of subcutaneous adipose tissue for different strain rates between 0.002 and 5700 s^−1^ and Grigoriadis investigated the material response of the heel fat pad for different strain rates up to 150 s^−1^ using a drop rig [27, 33]. Grigoriadis et al., however, tested the whole bulk of soft tissue beneath the calcaneus, which is composed of skin and adipose tissue, resulting in a combined mechanical response for both tissue types. In addition, both studies mainly focus on adipose tissue behaviour at different strain rates and do not further investigate the aspect of irreversible tissue deformation. For the development of tissue models capable of depicting blunt impact force responses for forensic applications, further studies on adipose tissues under compression-dominated impact load cases are required.

Hence, the aim of this study is (i) to characterise adipose tissue behaviour up to irreversible deformation, (ii) to relate this to the microstructural configuration, (iii) to quantify this deformation and (iv) to provide an analytical basis for computational modelling of adipose tissue under blunt impact.

## Material and methods

### Specimen preparation

Porcine subcutaneous adipose tissue is used as a biological model for human subcutaneous adipose tissue in this study. Samples consisting of skin, subcutaneous adipose tissue and the underlying muscle were resected from the abdomen of freshly slaughtered pigs (Swabian-Hall swine) from a local butcher and tested within 24 h after slaughter. Pigs were reared organically and had an age of approximately 8 to 9 months at the time of slaughter. For specimen preparation, the excised samples were cut to a rectangular shape with a size of approximately 60 mm × 60 mm. The tissue layers of skin and muscle were carefully removed with a scalpel (blade No. 22, Bayha, Tuttlingen, Germany) to create specimens that consisted only of the subcutaneous adipose tissue layer. Afterwards, the specimens were cut to a size of 50 mm × 50 mm using a custom-built punch (Wiedemann, Hechenwang, Germany) to create specimens of uniform width. Specimen thickness represented the original thickness of the subcutaneous layer between the skin and muscle tissue. The samples were moistened with 0.9% sodium chloride solution at regular intervals during the preparation process to avoid dehydration.

### Test setup

Drop tower experiments from different falling heights were performed to investigate the material behaviour of subcutaneous adipose tissue under blunt impact. A drop tube device was used to specify the conditions of the impact for the tests. The impactor was a custom-built hollow aluminium cylinder with an outer diameter of 36 mm, an inner diameter of 26 mm, a height of 200 mm and a weight of 0.918 kg (Wiedemann, Hechenwang, Germany). The drop height of the impactor could be continuously adjusted using a permanent electromagnet. Adipose tissue specimens were placed on a steel ground plate with dimensions of 100 mm × 100 mm × 20 mm. The side of the specimen that was connected to the skin before specimen preparation was facing towards the impactor.

**Fig. 1:**
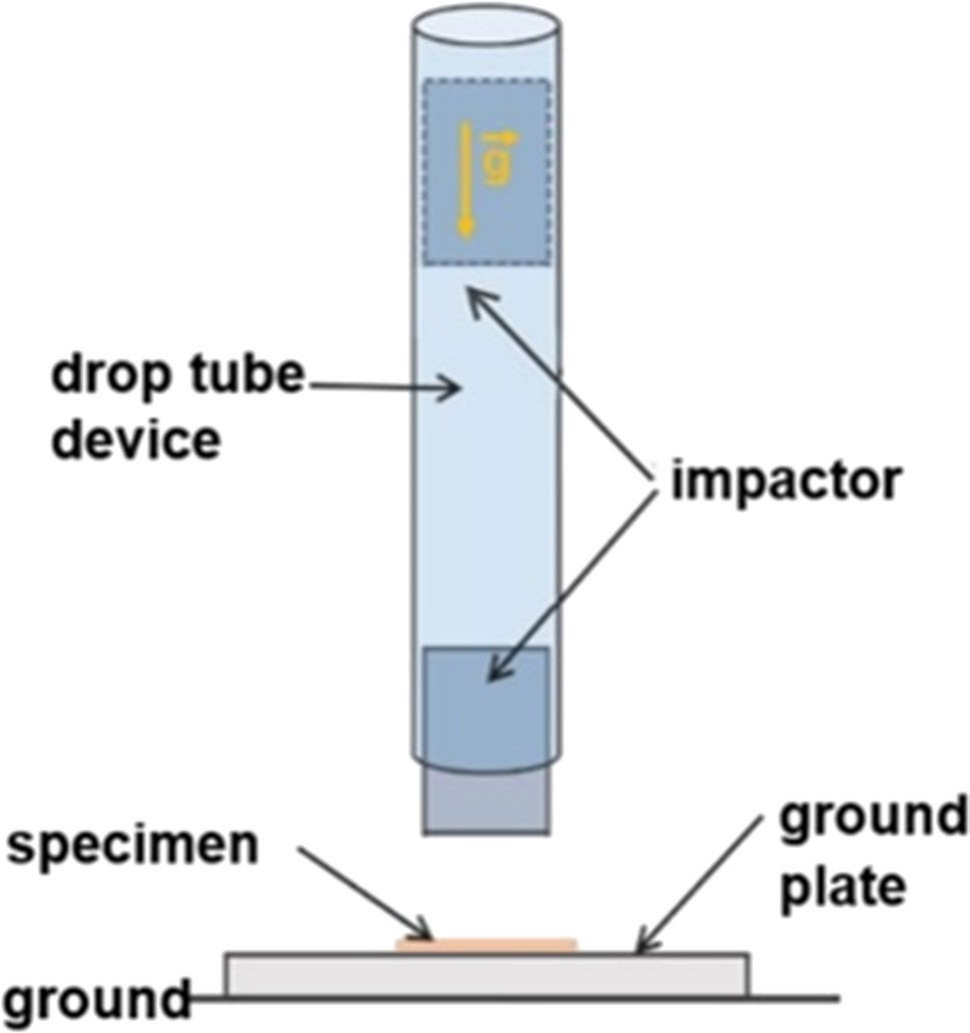
Drop test setup. Adipose tissue specimens were placed on a steel ground plate and impacted by a cylindrical impactor from different falling heights

Impactor acceleration during impact was recorded using an acceleration sensor with a range of ± 5000 g (EGCS3-D, Entran, New Jersey, USA), which was rigidly mounted inside the cylindrical impactor. Sensor output was transferred to a measuring amplifier (MV 3001, IHM Messtechnik, Neustadt, Germany) and the acceleration signal was recorded at a sampling rate of 50 kHz. Acceleration data was filtered with an SAE J211/1 low-pass filter at a channel frequency class of 600. Data filtering and comparison were performed in Hypergraph (version 2017.2, Altair, Troy, USA). Figure 1 depicts a schematic of the experimental setup.

### Testing parameters

Drop tests were performed from three different falling heights—4 cm, 8 cm and 30 cm—corresponding to impact velocities of 886 mm/s, 1253 mm/s and 2426 mm/s, respectively. Four specimens were tested for each falling height. In addition, four specimens were tested at a falling height of 8 cm using a different impactor geometry to investigate the influence of the impacting geometry on the material behaviour. For these tests, a similar impactor was used with a half-sphere instead of a flat cylindrical geometry. The radius of the half-sphere was 18 mm, total impactor weight amounted then to 0.941 kg. In addition, three tests for a falling height of 8 cm were conducted, where specimen dimensions were documented before testing and directly as well as 4 h after the impact to explore whether the deformation of the specimens after impact was permanent or not. Specimens were covered with towels soaked with 0.9% sodium chloride solution and moistened at regular intervals during the 4 h to avoid dehydration. In total, 19 drop tower tests were performed.

### Documentation of specimen geometry

Measurement of specimen dimensions was performed using photogrammetry. The adipose tissue specimen, resting on the steel plate used in the drop test setup, was placed onto a rotatory table in a light cube. Pictures of the specimen were taken at intervals of 10° during one full turn of the rotatory table. One set of pictures represented a 360° image of the specimen. A cube with an edge length of exactly 10 mm was placed next to the adipose tissue specimen to serve as a dimensional reference for correct scaling in the post-processing. Pictures were taken with a digital reflex camera (Nikon D3300, Nikon, Tokyo, Japan) and a zoom lens with an adjustable focal length between 18 and 55 mm (Nikon AF-S DX Nikkor 18–55 mm 3.5–5.6G VR 52 mm, Nikon, Tokyo, Japan) at a resolution of 24 MP. The following camera settings were used—focal length was set to 35 mm, shutter speed to 1/25 s and aperture to f/11. The camera was mounted on a tripod (TM2324, K&F Concept, Shenzhen, China) such that the axis of the lens exhibited an angle of 40° with respect to the specimen surface. The camera position remained fixed during the whole procedure. Photogrammetry of each specimen was performed before and directly after drop tower testing. The specimen remained on the steel plate throughout the whole process, i.e. photogrammetry before testing, drop tube testing and photogrammetry after testing, to avoid that its shape or position changes due to manual handling. Pictures were processed with the software Agisoft PhotoScan (version 1.1.4, Agisoft LLC, St. Petersburg, Russia) to reconstruct 3D models of the specimens in STL (standard tessellation language) format. For thickness measurement, the models were imported in 3matics (version 17.0, Materialise GmbH, Leuven, Belgium) and scaled to realistic dimensions using the cube as dimensional reference. After scaling, models of one specimen before and after impact were overlaid following a standardised procedure of translational and rotational operations. Dimensional differences of the model after impact were analysed in comparison to the original model and visualised by colours of a corresponding legend to illustrate specimen deformation after impact. The colour legend represents the dimensions of the impacted specimen compared to the original specimen in millimetres. Specimen thickness was evaluated by measuring perpendicularly to the underlying steel plate surface. Thickness was measured at five different locations—the approximate centre of the impactor impression and four equally distributed points with a distance of 9 mm (cylindrical impactor) or 1.8 mm (half-sphere impactor) to this centre point. By overlaying the models, thickness could be measured at approximately the same locations for both, the original and the impacted model. An average thickness for the specimen before and after impact was calculated, as well as the thickness change between the original and impacted model in percent.

## Results

### Falling heights

Table 1 summarises thickness data and peak acceleration, as well as the respective median values for each falling height, for the drop tests performed with a cylindrical impactor geometry. Specimen thickness varies between 2.1 and 9.4 mm, peak acceleration between 34.9 and 393.4 g and thickness change before and after testing from 11.5 to 58.3% for all tests.

**Table 1:** Thickness and acceleration data for different drop heights. The table lists specimen thickness before testing, specimen thickness after testing, thickness change for the specimen before and after testing and peak acceleration for each specimen, as well as the respective median values for each falling height (4, 8 and 30 cm)

Specimen	Thickness before testing (mm)	Thickness after testing (mm)	Thickness change (%)	Maximum acceleration (g)
4 CM #1	5.3	4.5	14.8	62.0
4 CM #2	5.4	4.8	11.5	63.4
4 CM #3	5.6	3.9	29.8	60.2
4 CM #4	9.4	7.7	18.6	34.9
4 CM median	5.5	4.7	16.7	61.1
8 CM #1	2.3	1.4	37.3	145.4
8 CM #2	3.6	2.4	33.4	133.0
8 CM #3	4.6	3.3	27.6	110.2
8 CM #4	6.7	5.4	19.6	87.0
8 CM median	4.1	2.9	30.5	121.6
30 CM #1	2.1	1.4	32.7	393.4
30 CM #2	3.7	2.0	45.9	269.2
30 CM #3	4.9	2.1	58.3	259.2
30 CM #4	5.4	3.7	31.5	210.7
30 CM median	4.3	2.0	39.3	264.2

**Fig. 2:**
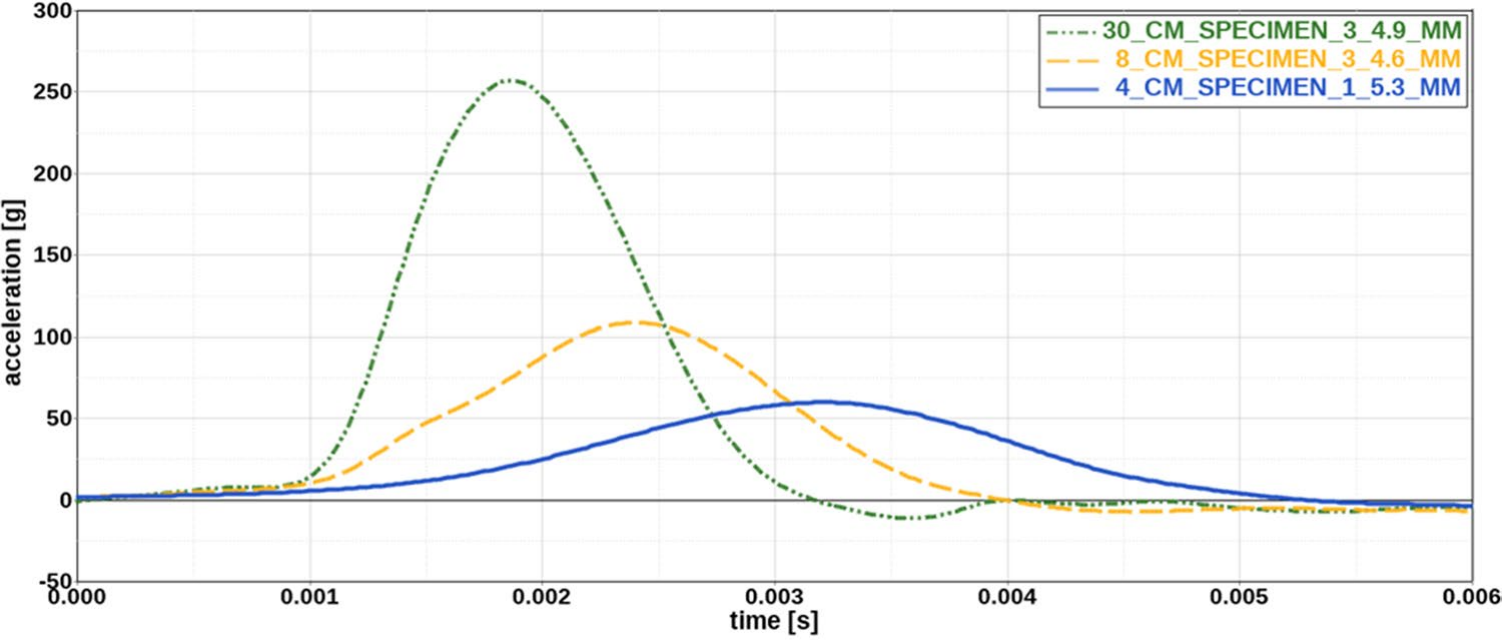
Comparison of acceleration curves for different drop heights. With increasing drop height, curve progression becomes steeper resulting in an increase in maximum acceleration and a decrease in impact time. Thickness of each specimen is indicated in the legend

**Fig. 3:**
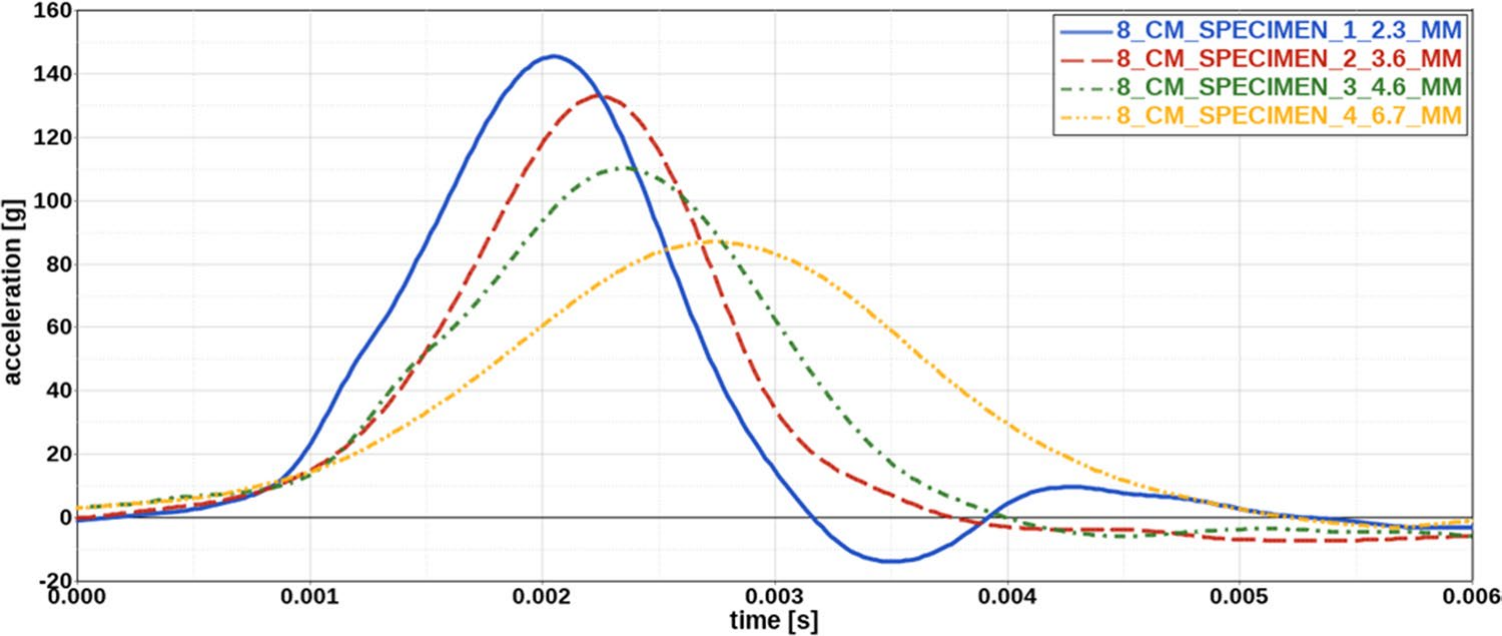
Influence of specimen thickness on acceleration curves. For increasing specimen thickness, peak acceleration decreases, whereas impact time increases. Thickness of each specimen is indicated in the legend

Figure 2 compares acceleration results for the specimens tested at the three different falling heights (4 cm, 8 cm and 30 cm). The main features of the acceleration-time curves are similar for the different drop heights. Curve progression shows a moderate slope at the beginning of the impact, followed by a steep increase in acceleration until the curve maximum is reached. Nevertheless, acceleration curves become steeper for higher impact velocities with increasing peak acceleration and decreasing impact time. This trend is also reflected by the median peak acceleration values for each falling height, which amount to 61.1 g for 4 cm, 121.6 g for 8 cm and 264.4 g for 30 cm. Relating acceleration curve results and specimen thickness for each falling height, it can be observed that acceleration curves are affected by specimen thickness with a trend to higher peak acceleration and shorter impact time for thinner specimens. Figure 3 illustrates these effects, depicting the acceleration curves for specimens with different initial thicknesses tested at a drop height of 8 cm as a representative example for all drop heights.

**Fig. 4:**
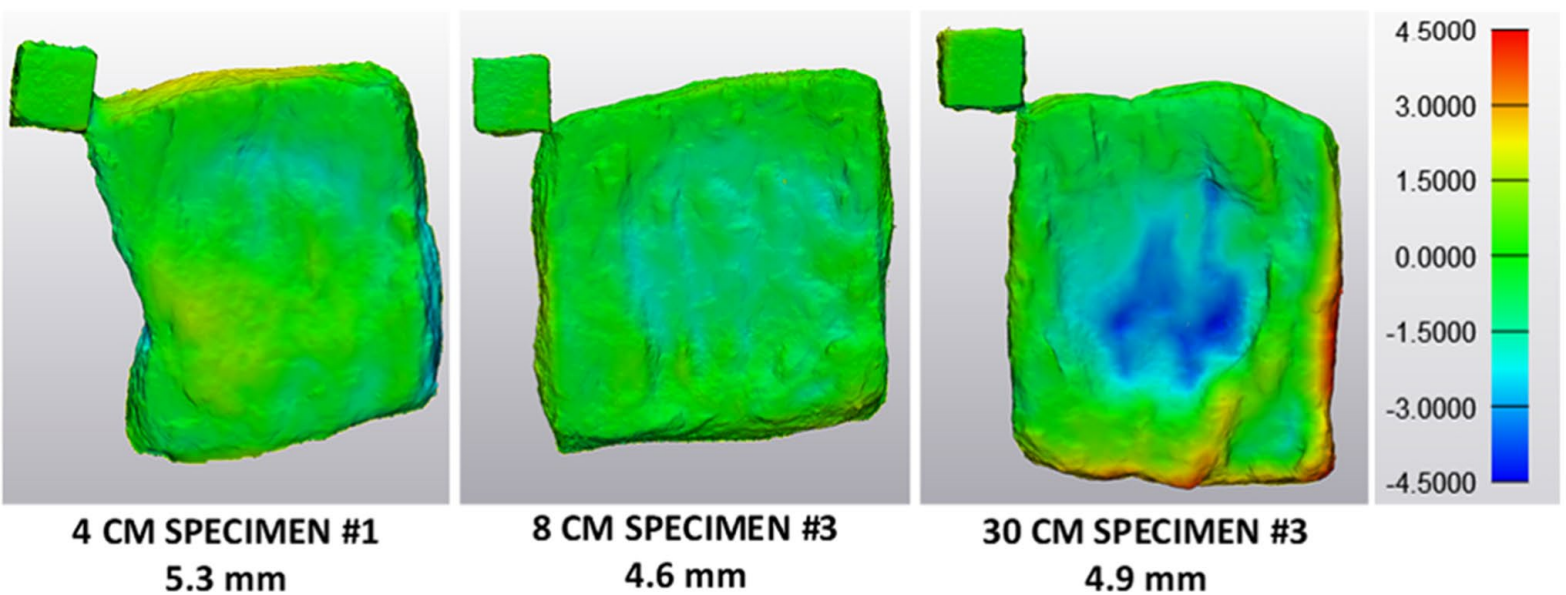
Comparison of specimen dimensions before and after impact for different falling heights. Deformation of the specimens after testing shows an indentation with the shape of the impactor geometry. This indentation becomes more extensive with increasing falling heights. The colour legend represents the dimensions of the impacted specimen compared to the original specimen in millimetres

Considering specimen deformation, the median thickness change between impacted and original specimen increases from 16.7% over 30.5 to 39.3% with increasing drop height. This is also reflected by Fig. 4, which compares specimen dimensions before and after impact for the three different falling heights. In general, deformation of the specimens results in a rather flat indentation with the shape of the cylindrical impactor geometry at the impact site. For a drop height of 4 cm, this indentation was hardly traceable by visual examination and could only be visualised by 3D analysis. With increasing drop height, the impression becomes more extensive, as can be seen in Fig. 4. Nevertheless, for none of the falling heights, a complete rupture within the specimen, i.e. an opening throughout the complete tissue thickness, could be detected after the impact.

### Impactor geometries

Table 2 summarises thickness data, peak acceleration and the respective median values for the specimens tested with a half-sphere impactor geometry from a drop height of 8 cm (corresponding to an impact velocity of 1253 mm/s). For specimens 1 and 2, the adipose tissue was completely ruptured at the impact location after testing, i.e. the tissue structure exhibited an opening throughout the whole tissue thickness, depicted by zero thickness after testing. For these two specimens, the impactor hit the steel plate during the corresponding experiments leading to high peak acceleration values that do not only reflect adipose tissue material behaviour but in part also that of the underlying steel plate. Results for specimens 3 and 4, in contrast, show a lower maximum acceleration in comparison as they were not completely ruptured during testing. This phenomenon seems to be linked with specimen thickness. Specimens 1 and 2 exhibit a thickness of 3.0 mm and 3.1 mm before testing, whereas specimens 3 and 4 show a thickness of 6.7 mm and 7.2 mm before testing. Median specimen deformation with respect to the original specimen thickness amounts to 78.7%.

**Table 2:** Thickness and acceleration data for a drop height of 8 cm for the half-sphere (HS) impactor geometry. The table shows specimen thickness before testing, specimen thickness after testing, thickness change for the specimen before and after testing and peak acceleration for each specimen, as well as the respective median values

Specimen	Thickness before testing (mm)	Thickness after testing (mm)	Thickness change (%)	Maximum acceleration (g)
HS 8 CM #1	3.0	0.0	100	314.2
HS 8 CM #2	3.1	0.0	100	296.5
HS 8 CM #3	6.7	3.1	53.7	71.8
HS 8 CM #4	7.2	3.1	57.5	36.6
Median	4.9	1.5	78.7	184.2

**Fig. 5:**
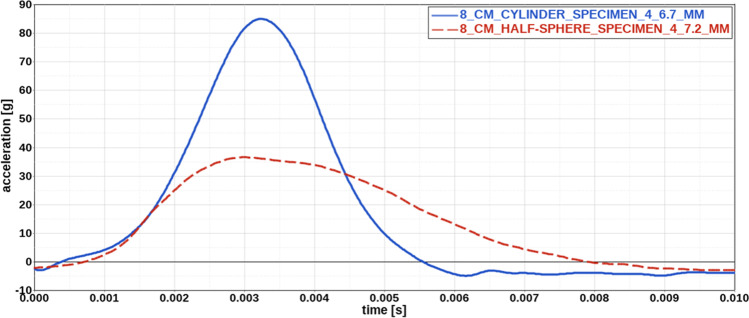
Comparison of acceleration curves for different impactor geometries. Acceleration curve for the cylindrical impactor geometry exhibits a steeper slope with a higher peak acceleration and shorter impact time than the curve for the half-sphere geometry. Thickness of each specimen is indicated in the legend

Figure 5 compares acceleration results for the cylindrical and the half-sphere impactor geometry at a drop height of 8 cm for specimens with a thickness of about 7.0 mm. The acceleration curve for the cylindrical impactor geometry is steeper, with a shorter impact time and a higher peak acceleration of 87.0 g compared to a peak acceleration of 36.6 g for the half-sphere geometry. This is not reflected by the median peak acceleration values, which amount to 121.6 g for the cylindrical and 184.2 g for the half-sphere geometry. However, it might be reasonable to leave results for specimens 1 and 2 impacted by the half-sphere geometry out for comparison, considering that these results reflect in part also steel behaviour. Median peak acceleration for specimens 3 and 4 only amounts to 54.2 g and is considerably below the median value for the cylindrical geometry at the same falling height.

**Fig. 6:**
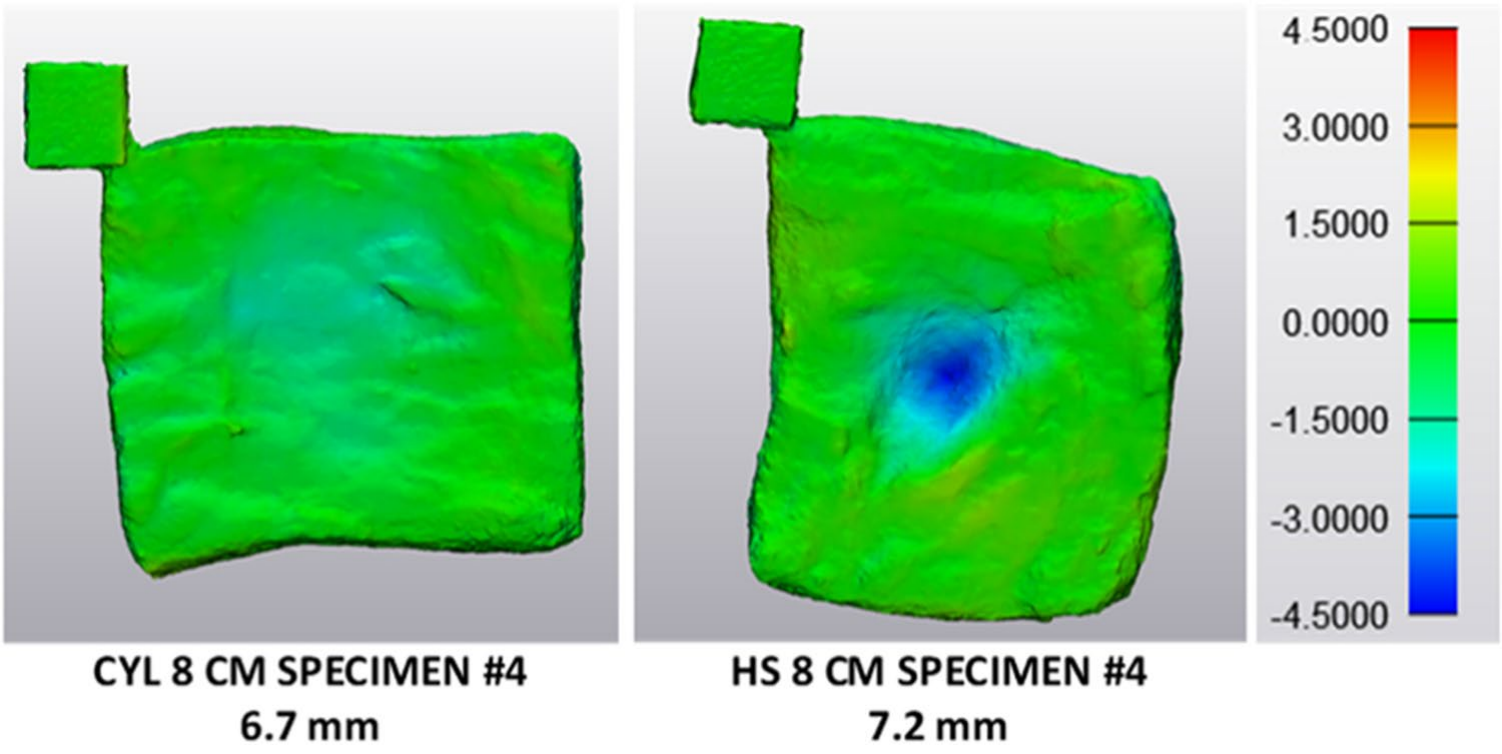
Comparison of specimen dimensions before and after impact for different impactor geometries. Deformation of the specimens tested with the half-sphere (HS) shows an indentation reflecting the half-sphere geometry. Indentation is deeper and more concentrated for the specimens impacted by the half-sphere than for specimens impacted by the cylindrical geometry (CYL). Colour legend represents the dimensions of the impacted specimen compared to the original specimen in millimetres

With 78.7%, the median specimen deformation for tests with the half-sphere geometry is relatively high compared to the value of 30.5% for tests with the cylindrical impactor at a drop height of 8 cm. It is even higher than the median specimen deformation for specimens impacted from a falling height of 30 cm by the cylindrical geometry, which amounts to 39.3%. Figure 6 compares specimen deformation for the different impactor geometries for a specimen thickness of around 7 mm. Deformation of the specimens tested with the half-sphere geometry shows an indentation reflecting the shape of the half-sphere at the impact site. In contrast to the experiments with the cylindrical geometry, the indentation is not flat but rather shows the shape of a crater and was always traceable by visual examination. Generally, indentations on specimens impacted by the half-sphere are deeper and more concentrated than indentations on the specimens impacted by the cylindrical geometry. Even a complete rupture within two specimens could be detected after testing with the half-sphere geometry by visual examination as well as by 3D analysis.

### Recovery of specimen deformation

**Fig. 7:**
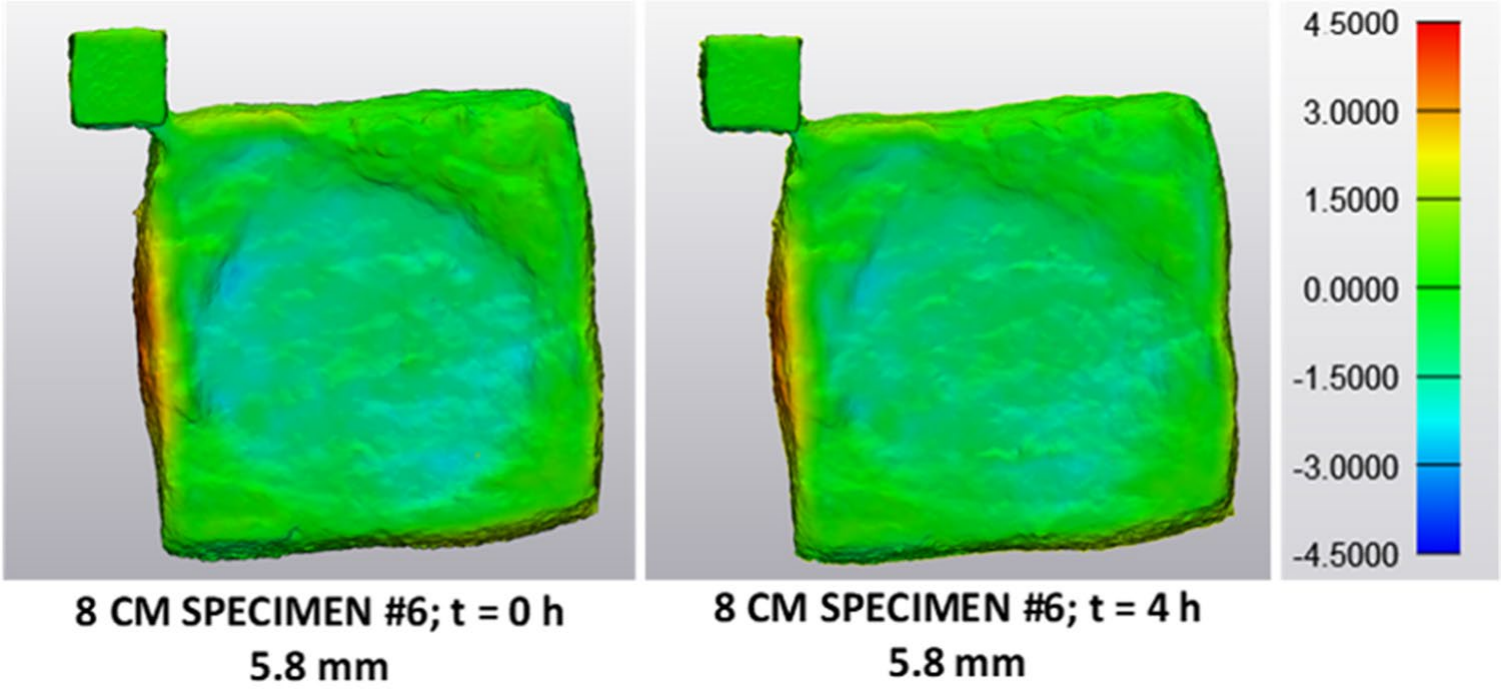
Comparison of specimen geometries before and after impact, directly and 4 h after testing. Illustrated is the dimensional difference of the models directly after impact and 4 h after impact for specimen 6 in comparison to the model before impact. Four hours after the impact, a small part of the specimen deformation is recovered compared to the deformation directly after impact, whereas the greater part of the specimen deformation remains. Colour legend represents the dimensions of the impacted specimen compared to the original specimen in millimetres

Table 3 lists specimen thickness, thickness change of the impacted specimen in relation to the original specimen directly after the impact and 4 h after the impact, as well as the thickness recovery of the specimen four hours after the impact. For all investigated specimens, thickness change directly after testing is higher compared to the thickness change four hours after testing. This means that part of the deformation is recovered during this period. However, with 3.1%, the median amount of recovery is quite small compared to the median amount of deformation of 25.1%. Thus, even 4 h after testing, the greater part of the specimen deformation remains. This is also reflected in Fig. 7, which compares specimen dimensions before and after impact for specimen 6 at the different time points.

**Table 3:** Thickness change between original and impacted specimen at different time points after impact. The table lists the original thickness of the specimen, the thickness change between original and impacted specimen in percent directly after the impact and 4 h after the impact, the thickness recovery of the specimen 4 h after the impact and the respective median values

Specimen	Thickness before (mm)	Thickness change (*t* = 0 h) (%)	Thickness change (*t* = 4 h) (%)	Thickness recovery (*t* = 4 h) (%)
8 CM #5	3.4	36.8	33.7	3.1
8 CM #6	5.8	24.7	21.4	3.3
8 CM #7	8.3	27.3	25.1	2.2
Median	5.8	27.3	25.1	3.1

## Discussion

### Photogrammetry approach

A special focus was set on thickness measurement of the adipose tissue specimens during the experimental procedure. The measurement of adipose tissue dimensions presents a challenge as the tissue is so soft that its shape can be already deformed by slight touching. Thus, manual measurement methods, such as measurement with callipers, might already deform the specimen and lead to inaccurate measurement results. Therefore, in this study, photogrammetry was used to reconstruct a 3D model of each specimen geometry before and after impact. Thickness was not measured on the physical specimen, but on the 3D models allowing a contactless measurement of the specimen. Thus, measurement errors due to specimen deformation by manual handling were minimised. After overlaying the 3D models of a specimen before and after testing, dimensional differences between the models were analysed and illustrated resulting in an improved visualisation of specimen deformation after impact. Deformations that could not be detected by visual examination could be depicted with this method. The improved visualisation permitted a thickness measurement of the specimen directly at the location of impact, which was not always located exactly in the specimen centre. This was important, as specimens were not of uniform thickness, but represented the original shape of the subcutaneous adipose tissue layer, which varied in thickness. By evaluating specimen thickness before and after testing at the site of impact, specimen deformation was quantified. Besides, 3D models of the specimens before testing may be used as geometry input for subsequent computational modelling, whereas 3D models of the specimens after testing will be useful in the future to develop failure and damage models for adipose tissue. 3D model geometry after impact from experiment and simulation can be compared to investigate, whether the failure and damage models are able to reproduce a similar deformation of the adipose tissue. Another advantage of photogrammetric documentation is that it can be performed rather quickly during the actual experimental procedure. It takes around 5 min to take the pictures needed for the reconstruction of one specimen. This is important, as too long measurement periods would result in dehydration of the specimens. The method also enables the measurement of specimen geometry at any time after testing, which can be useful to recheck existing or perform new measurements.

### Experimental setup

One objective of the drop tower tests was to characterise subcutaneous adipose tissue behaviour under intermediate strain rates and high deformations up to the regions, where tissue behaviour becomes irreversible. Different falling heights are analysed in this paper to investigate the influence of strain rate on adipose tissue behaviour. In addition, falling heights were chosen in a way that different deformation stages of the adipose tissue specimen were produced. For the lowest falling height of 4 cm, only a small amount of specimen deformation was detected. Falling heights of 8 cm and 30 cm were chosen to produce specimens with intermediate and high irreversible tissue deformation. Besides, different impactor geometries were tested to examine the influence of different loading scenarios on material behaviour—a scenario representing distributed loading using a cylindrical impactor and a scenario representing a more concentrated loading using a half-sphere impactor. Another purpose of this study was to design experiments that can be applied as an analysis setup for computational modelling of adipose tissue. Thus, experimental boundary conditions do not reflect a realistic blunt impact scenario, where adipose tissue is covered by skin and compressed against bone, not a steel plate. However, experiments of the present study are intended to characterise the mechanical behaviour of subcutaneous adipose tissue only, not as a model for soft tissue injury under blunt impact in general. The experimental setup was designed in a way that the only unknown is the material behaviour of the adipose tissue specimen. Therefore, the layers of skin and muscle tissue were resected for the experiments as they exhibit different material behaviour than adipose tissue [45–49]. Results reflecting a mixed behaviour of the three different tissue layers are of little use for the purpose of analysing or modelling adipose tissue behaviour only. For a similar reason, steel was used as material for the ground plate, as materials, that can be used as a bone substitute, such as wood, exhibit themselves complex material behaviour, whereas material modelling of steel is quite straightforward. The developed experimental setup is rather simple with well-defined boundary conditions and can therefore provide a reasonable analysis basis for computational modelling of adipose tissue under blunt impact. And, it provides further insights on the material behaviour of adipose tissue in addition to testing under pure shear, uniaxial compression or tension.

### Experimental results

Results for impactor acceleration showed a dependency on specimen thickness. In general, impactor acceleration decreased and impact time increased with higher tissue thickness, indicating a damping effect of the subcutaneous adipose tissue. This is consistent with the fact that one of the main functions of subcutaneous adipose tissue in the human body is to serve as a shock-absorbing layer [20]. Acceleration curves showed a distinct subdivision in an initial phase exhibiting a shallow gradient and a subsequent regime of steep acceleration increase. This is similar to the shape Comley and Fleck observed for the stress–strain response of porcine subcutaneous adipose tissue under uniaxial compression, which was characterised by a moderate slope for low compressive strains, followed by a rapid increase in stiffness for strains above 25% [27].

Comparing the acceleration results for different impactor geometries, it is interesting that peak acceleration values for specimens tested with the half-sphere geometry are lower compared to the peak acceleration values for specimens tested with the cylindrical geometry at the same falling height. This does not hold for the two specimens that were completely ruptured during testing; however, acceleration results for these tests do not only reflect the material behaviour of adipose tissue but partly also that of steel. The difference in acceleration results between the different impactor geometries might be due to different deformation behaviours for both geometries. Deformation for specimens tested with the cylindrical geometry is quite low (median thickness change of 30.5%) compared to the deformation of that tested with the half-sphere geometry (median thickness change of 78.7%). Therefore, the lower peak acceleration results might be related to a higher amount of energy dissipation in the adipose tissue specimens impacted by the half-sphere geometry due to a greater extent of damage or failure.

For falling heights of 8 cm and 30 cm, impact on the adipose tissue resulted in an indentation reflecting the shape of the impactor for both, cylindrical and half-sphere geometry. This behaviour is rather different to that of several other soft tissues, like skin, tendons or arteries, where failure leads to a crack-like laceration or rupture of the tissue [6, 49–52]. In addition, the greater part of the observed specimen deformation was irreversible as shown by analysis results for specimen deformation directly and 4 h after impact. It is supposed that changes within the microstructure of the adipose tissue during impact lead to this permanent deformation. The period of 4 h was based on the results of a study of Geerligs et al. who investigated the long-term response of porcine subcutaneous adipose tissue subjected to small shear straining [42]. They observed an increase in the shear modulus of the adipose tissue of about one order of magnitude at loading times between 250 and 1250 s, which they attributed to structural changes within the adipose tissue. They found this increase to be reversible, when the tissue can recover for a period of 3 h after loading. It is assumed that mechanisms, which lead to a recovery of the adipose tissue deformation in our experiments, would occur on a similar time scale.

**Fig. 8:**
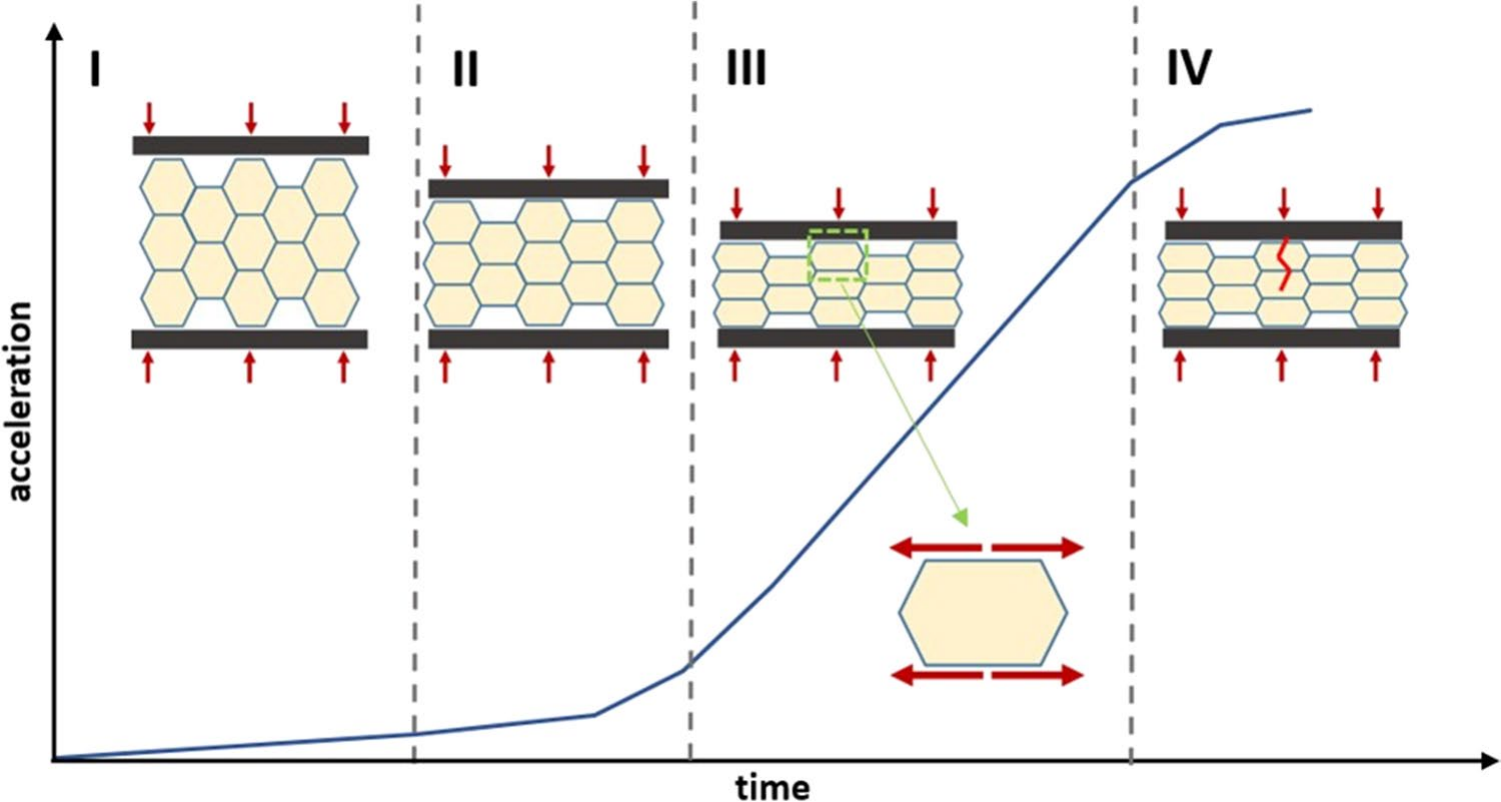
Hypothesised deformation mechanism of subcutaneous adipose tissue under blunt impact in four phases: I, behaviour mainly governed by the viscous lipid inside the adipocytes (shallow slope of the acceleration curve); II, compression of the lipid, adipocyte cell walls stretching perpendicular to the loading direction, activation and reorientation of collagen fibrils in the reinforced basement membrane, collagen fibres recruitment; III, steep gradient, tensile loading of the collagen fibres of the extracellular adipose tissue matrix; IV, the reinforced basement membrane of single adipocytes is supposed to rupture in the region of highest membrane strain. Adipocyte rupture site as an example only; interlobular septa not depicted

Considering the aforementioned observations, it seems that subcutaneous adipose tissue behaviour is strongly linked to its microstructural architecture. Similar to the studies of Comley and Fleck, it is hypothesised here that adipose tissue behaviour resembles that of a fluid-filled closed-cell foam with flexible cell walls, interwoven by a network of fibres [21, 27]. It is supposed that in the initial phase of the impact, mechanical behaviour is mainly governed by the viscous lipid inside of the adipocytes reflecting the initial shallow slope of the acceleration-time curves. During further loading, compression of this nearly incompressible lipid leads to a stretching of the adipocyte cell walls in the plane perpendicular to the loading direction. This deformation mechanism for adipocytes was observed by Comley and Fleck, who performed a confocal microscopical analysis of porcine subcutaneous adipose tissue subjected to uniaxial compression [21]. Also, Seyfi et al., who developed a micromechanical model of adipose tissue consisting of adipose tissue extracellular matrix and lipid droplets representing the adipocytes, could notice a similar deformation mechanism during simulation of compressive loading [53]. The spherical shaped adipocytes develop a more elliptical shape; the reinforced basement membrane, surrounding each adipocyte, is stretched and, thereby, subjected to tension. It is supposed that this results in the activation and reorientation of collagen fibrils in the reinforced basement membrane in the direction of the described tensile loading. During this phase of the impact, collagen fibres start to carry part of the load imposed on the specimen corresponding to the transition between the shallow and the steep slope in the acceleration curves. This process might be compared to the behaviour of collagen fibres in the dermis during uniaxial tensile loading [51, 54, 55]. Only, that in subcutaneous adipose tissue not the fibres of a mesh-like network intervening the tissue matrix, but the fibres in the membrane surrounding each cell are activated. In a similar manner, the collagen fibres of the interlobular septa network might be stretched and activated and, thus, contribute to this behaviour. In the third phase, depicted by the steep gradient of the acceleration curves, principal mechanical behaviour is governed by progressive tensile loading of the collagen fibres of the extracellular matrix. If the adipose tissue is compressed even further, it is assumed that the reinforced basement membrane of single adipocytes ruptures in the region of the highest membrane strain (compare Fig. 8) and the viscous lipid leaks out of the cell into the tissue space.

This effect would explain the irreversible deformation that was observed for the adipose tissue specimens after testing in this study. An assumption that is further supported by findings of Comley and Fleck—in scanning electron microscopy analysis of the crack zone of subcutaneous adipose tissue after trouser tear testing—they observed that the reinforced basement membrane of single adipocytes was ruptured [26]. The theory that tensile effects of the extracellular matrix play an important role for adipose tissue mechanics under compression is consistent with results from another work of Comley and Fleck, who found a similar stress–strain response for subcutaneous adipose tissue in uniaxial tension and compression [27]. In addition, computational models for adipose tissue considering the tissue microstructure and the interaction between adipocytes and extracellular matrix support the findings that this interaction contributes to the observed material response [53, 56, 57]. Histological analysis of adipose tissue specimens after impact testing might help to further investigate the hypothesis described above and the role of reinforced basement membrane and interlobular septa during blunt impact. Figure 8 summarises this theory by relating the microstructure of the adipose tissue to different regions of a schematic acceleration-time curve representative of the results of this study.

### Limitations of the study

There are several limitations of the present study. First, porcine subcutaneous adipose tissue was used as a biological model for human subcutaneous adipose tissue due to reasons of ethics and availability. Also, in several other studies, porcine adipose tissue is used as a surrogate for human adipose tissue, as the composition and structure of porcine adipose tissue in general are similar to that of humans [26, 27, 58]. Pigs are even used as models for in vivo studies to investigate wound healing or adipose tissue remodelling due to their similarity to humans [59, 60]. Adipocytes in porcine subcutaneous adipose tissue exhibit a diameter of ca. 80 μm like the adipocytes in human subcutaneous adipose tissue, the viscous properties of the lipid inside the adipocytes are similar and the extracellular matrix also exhibits a porous structure comprised by a reinforced basement membrane in combination with interlobular septa [20, 21]. However, porcine subcutaneous adipose tissue is more compact and the interlobular septa are thicker compared to human tissue [61]. Due to these differences, material behaviour is expected to differ for porcine and human subcutaneous tissue. With a value of about 2.5 kPa, Sommer et al. found the Cauchy stress of human abdominal adipose tissue at a tensile stretch of 15% to be about 25 times higher than the Cauchy stress of 0.1 kPa observed by Comley and Fleck for porcine subcutaneous adipose tissue at the same tensile stretch [27, 40]. Sun et al., in contrast, tested human abdominal and porcine dorsal adipose tissue under uniaxial compression and simple shear at a strain rate of 3 s^−1^ and found that the porcine tissue behaved stiffer for both loading scenarios [31]. Nevertheless, the microstructure of human and porcine subcutaneous tissue is quite similar and, as material behaviour is supposed to be strongly linked to the microstructure of the tissue, porcine subcutaneous adipose tissue seems to be an acceptable model for human tissue. Material behaviour for human adipose tissue may differ in quantity and also to some extent in quality, but it is expected that the principal material mechanics are similar for human and porcine subcutaneous adipose tissue.

Specimen thickness was not uniform in this study but represented the original thickness of the subcutaneous adipose tissue layer between the skin and muscle tissue. As adipose tissue is very soft and deforms easily, cutting specimens to a uniform thickness is hardly feasible for specimens with dimensions of 50 mm × 50 mm without freezing the adipose tissue specimen. And even for frozen specimens, a clear cut at a certain height is difficult to perform and uniform thickness is not guaranteed. As freezing might also influence tissue structure or material properties of the adipose tissue, this study was conducted without any freezing of specimens (see also Alkhouli et al., Geerligs et al. and Roehm [20, 41, 58]).

Another drawback of the setup is that testing was performed at room temperature only, whereas adipose tissue exhibits a temperature of around 37 °C in the human body. This difference in temperature might influence the mechanical response of the adipose tissue. Indeed, Comley and Fleck tested porcine adipose tissue under compression at room temperature and at 37 °C and could not find significant differences in material behaviour [27]. Geerligs et al., however, observed a strong temperature dependence for the shear modulus of porcine adipose tissue [41]. It is supposed that temperature affects primarily the quantity and not the quality of adipose tissue behaviour as microstructural architecture should not change dramatically with temperature, at least as long as it does not exceed the regime where components of the tissue start to denature. However, it would be preferable to repeat some drop test experiments at a temperature of 37 °C, although this is rather difficult to realise for such an experimental setting.

## Conclusions and outlook

The present study describes the mechanical characteristics of subcutaneous adipose tissue by providing experimental data for adipose tissue behaviour under blunt impact up to high and irreversible deformations. Tissue deformation is quantified by analysing the dimensional differences of 3D models of the specimens before and after impact testing. It is shown that part of the deformation is permanent. A theory relating the observed material behaviour to the microstructural architecture of the subcutaneous adipose tissue is developed. The theory states that adipose tissue behaviour in the initial impact phase is mainly governed by the viscous lipid inside the adipocytes, whereas further loading leads to activation and reorientation of collagenous structures in the extracellular matrix due to tensile effects. Irreversible tissue deformation is attributed to a rupture of single adipocytes and a subsequent leakage of the viscous lipid out into the tissue space. The proposed experimental setup in combination with the presented results provides a basis for computational modelling of adipose tissue behaviour, which can assist in evaluating the soft tissue influence on skeletal trauma for blunt impact scenarios.

In future work, the herein proposed theory will be further investigated using histological methods to analyse the deformation of the adipose tissue microstructure in blunt impact loading. Besides, the experimental drop test setup will be transferred to a computational model to further investigate subcutaneous adipose tissue behaviour under blunt impact using numerical methods.

## Data Availability

The datasets generated during and/or analysed during the current study are available from the corresponding author on reasonable request.
